# Pharmacist-Led Education for Final Year Medical Students: A Pilot Study

**DOI:** 10.3389/fmed.2021.732054

**Published:** 2021-09-23

**Authors:** Sophie Mokrzecki, Tilley Pain, Andrew Mallett, Stephen Perks

**Affiliations:** ^1^College of Public Health, Medical and Veterinary Sciences, James Cook University, Townsville, QLD, Australia; ^2^Pharmacy Department, Townsville University Hospital, Townsville, QLD, Australia; ^3^Allied Health Department, Townsville University Hospital, Townsville, QLD, Australia; ^4^College of Medicine and Dentistry, James Cook University, Townsville, QLD, Australia; ^5^Institute of Health Research and Innovation, Townsville University Hospital, Townsville, QLD, Australia

**Keywords:** pharmacist, medical education, medical students, prescribing skills, drug prescriptions, prescriptions, drug legislation

## Abstract

**Background:** Prescribing is a core skillset for medical officers. Prescribing errors or deficiencies can lead to patient harm and increased healthcare costs. There is an undefined role for pharmacist-led education to final year medical students to improve prescribing skills.

**Aim:** Assess if pharmacist-led education on prescription writing improves the quality and safety of final year medical students' prescribing skills.

**Method:**

***Participants and Intervention:*** Final year medical students were randomised into tutorial (TG) or non-tutorial groups (NTG) and assessed pre- and post- intervention. TG received education by a clinical pharmacist and pharmacy educator using case-based learning. NTG received no additional training as per usual practice. Following the pre-test, all students completed a 3-week tertiary hospital medical ward placement. Students completed the post-test following placement and after the TG participated in the intervention.

***Student Assessment:*** Assessment included writing Schedule 4 (S4, prescription only), Schedule 8 (S8, controlled drug), S4 streamline (S4SL), and Mixed case (S4 and S8) prescriptions.

**Results:** At baseline, there were no significant differences between TG and NTG for overall scores or proportion of passes. Post intervention scores significantly improved in TG (*p* = 0.012) whereas scores significantly decreased in the NTG (*p* = 0.004). The overall proportion of passes was significantly higher in the TG than NTG (*p* < 0.001).

**Conclusion:** Education by a clinical pharmacist improved short-term prescribing skills of final year medical students in this study. Students learning primarily experientially from peers and rotational supervisors showed decreased prescribing skills. We propose pharmacist-led education on prescription writing should be further evaluated in larger studies across more student cohorts and for longer periods of follow up time to clarify whether such an educational model could be included in future medical school curricula.

## Introduction

Medications are the most common health intervention worldwide (1). In Australia, almost 300 million prescriptions are covered by the Government per year under the Pharmaceutical Benefit Scheme (PBS) or Repatriation Pharmaceutical Benefit Scheme (2). If medication prescriptions are documented incorrectly or unclearly, this may lead to substantial patient harm (1, 3). Pharmacists are specifically educated and trained in the many aspects of safe and legal prescribing. This structured education means pharmacists are well-placed to provide education to future and current prescribers.

In 1994, the World Health Organization (WHO) published the Guide to Good Prescribing (GGP), where in 2001 the Teacher's Guide to Good Prescribing followed. The GGP is a 6-step model for rational prescribing aimed at undergraduate medical students and their teachers. Many places around the world have based their medical student or graduate learning and teaching on this model, including; the Netherlands, Canada, Spain, and Turkey (9). The National Prescribing Service (NPS) in Australia online modules on prescribing standards were developed based on the GGP. This web-based interactive prescribing module outlines competencies required to prescribe medicines (10). The NPS module is not compulsory in Australian medical school curriculum, however it must be completed by medical interns prior to working in a Queensland Health facility. Many online modules do not individually assess each legal component of a prescription or allow for interaction with an educator. The relative and potentially significant role of subsequent experiential learning is neither captured nor clarified. The UK have developed a compulsory online national Prescribing Safety Assessment (PSA) for final-year medical students based on a similar framework (10-step) (9). The differences in the delivery of medical education within and between European countries could impact students results on the PSA, thus supporting EACPT suggestion to create a uniform core curriculum for European medical schools (7).

In Australia, medical officers can prescribe once registered by the Australian Health Practitioner Regulation Agency and are bound by the Medical Board of Australia's Code of Practice (4). The Australian Medical Council sets standards for assessment and accreditation of primary medical programs. The Graduate Outcome Statement stipulates that upon entry into professional practice medical practitioners should prescribe medications safely and effectively (5, 6). The Medical Board of Australia's Code of Practice simply states that doctors must comply with State and Territory legislation (4). Frameworks for attainment of these regulatory requirements are somewhat implied though still unclear, including education for safe and legal prescription writing within medical degree programs for future medical practitioners to write prescriptions compliant with Australian legislation (5). Similarly, in most European countries, junior doctors are expected to have the baseline knowledge and skills as learnt in medical school in order to write prescriptions effectively and safely (7). The European Association of Clinical Pharmacology and Therapeutics (EACPT) aims to promote high professional standards in prescribing medications (8). However, like Australia, the methods of how this is achieved is unclear.

Interns and junior doctors write the highest proportion of medication prescriptions in hospital settings globally and it is therefore highly desirable for them to become proficient prescribers (3, 7, 11, 12). However, prescriptions written by medical interns may not be of high quality and may contain errors due to a multitude of factors (3, 7, 11–15). Prescribers in Australia must understand and follow the legal requirements of a prescription using state and territory guidelines; for example, in Queensland, the Health Drugs (and Poisons) Regulation (HDPR), 1996. There is potential jurisdictional incongruence however, as the Australian Curriculum framework for Junior Doctors only stipulates that junior doctors must document a medication prescription accurately (16). The framework implies but does not outline core competencies to safely prescribe, nor does it stipulate the need to adhere to local legal requirements.

It is essential we provide medical graduates with the skills needed to write a safe and legal prescription effectively prior to graduating. This study aims to assess if a pharmacist-led education session on prescription writing for final year medical students improves their subsequent short-term prescribing skills regarding safety and legality requirements.

## Methods

### Participants

All James Cook University (JCU) final year (sixth year) medical students in third term (June 18th to August 24th, 2018) at Townsville University Hospital (TUH) were invited to participate (*N* = 33). Medical students at other JCU medical student training sites, from other universities, or in years one to five were excluded. Other students from terms one, two and four were excluded due to time constraints of the study. Final year medical students were recruited as they are anticipated to transition into their internship within the next year, at which time they will apply medication prescribing skillsets. Therefore, it is predicted they would be more engaged in the content given approaching requirements for its application. All participants provided written informed consent and were advised they could withdraw at any time. Participants were randomly allocated into either non-tutorial group (NTG/control, standard education provided via university) or tutorial group (TG, provided an additional education session by a pharmacist). Randomisation was performed using Microsoft Excel randomisation tool.

**Figure 1:**
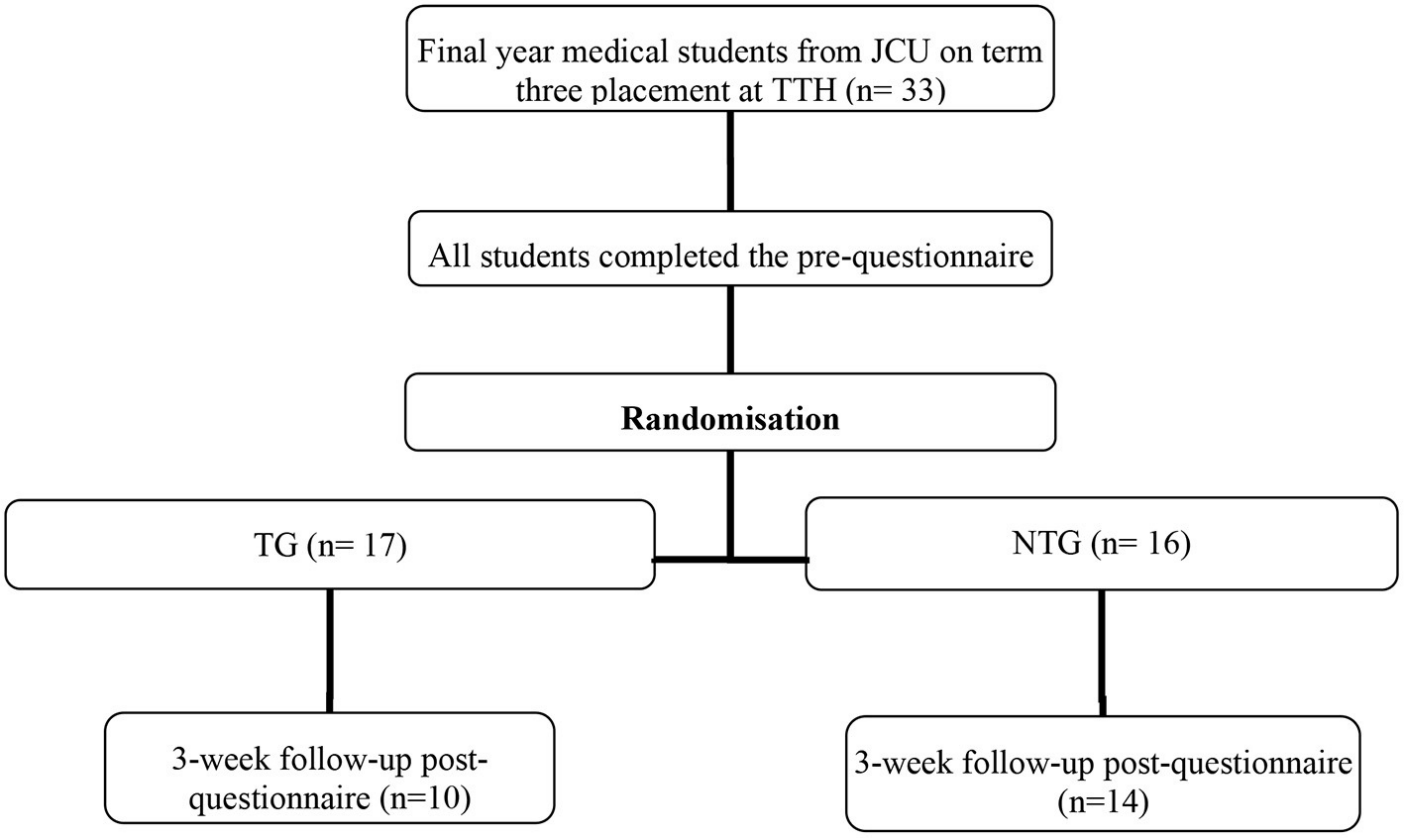
Research design and participant flow. N. B. Students in the TG (*n* = 7) and NTG (*n* = 2) were lost to follow-up due to placement requirements.

The overarching research design and participant flow diagram is presented in Figure 1.

### Intervention

One month after orientation week, participants in the TG received one 1.5-h structured education session delivered by a clinical pharmacist and pharmacy educator. Session content included legal and safety requirements of writing prescriptions. Resources were demonstrated to the students, including HDPR 1996, PBS, Australian Medicines Handbook, Therapeutic guidelines and Monthly Index of Medical Specialties. The education format was case-based learning (CBL) which provided students with an opportunity to learn relevant material and apply knowledge in different situations (17). During the session, students were given sample questions to practice writing scripts and encouraged to engage with the educators.

### Control

Participants in the NTG completed the pre- and post-questionnaires but were provided with no additional prescribing education, by a pharmacist or otherwise, as per the standard educational practice employed for their medical ward rotation. Both groups were likely to receive prescribing mentoring during their medical ward rotation by supervising medical staff. Neither the TG nor NTG were encouraged by the educating pharmacists to seek further information on prescription writing. The TG were not provided with hard-copy materials outside of the education session. Students may have undertaken their own self-directed learning; however, this variability was not assessed in this study.

### Student Assessment

Four types of cases generating five medication prescriptions were assessed. Cases were: Schedule 4 (S4), prescription only medication; Schedule 8 (S8), controlled drug; Schedule 4 streamline (S4SL); and a combined S4 and S8 case (mixed case where students were required to write two prescriptions). During the development phase of the assessment, each case was reviewed by pharmacy staff and an early career prescribing medical officer to determine the appropriateness. Different cases were used in the pre- and post-test, however both with the same types of cases generating five medication prescriptions.

Each prescription was assessed against compliance to 17 types of errors (see Appendix 1). However, the total score using the 17 legal and safety criteria for all four cases together was 70. The data collection tool allowed all errors to be identified and more than one error accounted for per case. The two educating pharmacists independently assessed the prescriptions and, where necessary, discussed to arrive at consensus for final marks.

### Statistical Analysis

Two levels of analyses were performed. One level looked at the number of errors on each prescription type (case). The other assessed an overall pass or fail of the entire case. For example, a student may receive a total mark of 66 out of 70, but if the prescription doesn't contain all the legal requirements according to the HDPR, in this study it was considered a fail for that case. The rationale for this type of assessment is that a pharmacist cannot legally dispense the prescription.

Data was collated in Microsoft Excel and imported to IBM SPSS Statistics (Version 25, IBM Corporation) for statistical analysis. An independent samples *t*-test was used to compare the overall change scores. Mann–Whitney *U*-tests were used to compare change scores for individual cases (the variables were not normally distributed so non-parametric tests were used) and to compare the overall number of cases passed. Mann–Whitney *U*-tests were also used to investigate differences in number of cases passed, post-test compared to pre-test, between the TG and NTG. Paired *t*-tests were used to analyse differences between pre-test total score and post-test total score within each group.

### Ethics

Townsville Hospital and Health Service Human Research and Ethics Committee approved this study (HREC/18/QTHS/142) and it was endorsed by the JCU Townsville Ethics Committee. Site Specific Approval was granted to conduct the study at TUH with final-year medical school students from JCU. The College of Medicine at JCU and Medical Placement coordinators at TUH provided approval and support for this study. All final year medical students (from terms one to four in 2018) were provided the opportunity to receive the education session after the pilot study was completed, meeting ethical standards of TUH HREC.

## Results

There were no significant differences at baseline for the scores or proportion of passes between TG and NTG.

Pre-test characteristics showed 25 students were aged 22–25 years and 6 students were over the age of 26, compared to 19 and three, respectively, post-test (two student did not answer both pre- and post-test). Three students had trained or worked in a health profession prior to their medical degree. One of these students was randomised to TG, and the other two were lost to follow-up.

### Overall Score

Students in the TG performed better overall in the post-test compared the NTG. Paired *t*-tests demonstrated the TG group significantly improved their score from pre- to post-test whereas the NTG overall score significantly decreased (Table 1).

**Table 1:** Mean and standard deviation of pre- and post-test scores for NTG and TG.

	**Pre-**	**Post-**	**Difference**	***P*-value**
NTG	61.8 ± 5.2	58.0 ± 5.3	−3.8 ± 4.7	0.012
TG	61.2 ± 6.0	66.6 ± 4.0	5.5 ± 4.5	0.004

### Individual Case Scores

The mean individual case scores for all prescription types significantly improved in the TG, while NTG scores significantly decreased (Table 2).

**Table 2:** Mean individual case scores pre- and post-test for the TG and NTG.

**Case**	**Group**	**Pre-test (mean ± sd)**	**Post-test (mean ± sd)**	**Mann–Whitney U-statistic**	***p*-value**
S4	TG	9.90 ± 1.10	10.80 ± 0.42	20.0	0.002
	NTG	10.21 ± 0.98	9.64 ± 0.75		
S8	TG	18.05 ± 2.52	19.90 ± 1.79	17.0	0.002
	NTG	17.96 ± 1.65	17.14 ± 2.03		
S4SL	TG	10.50 ± 0.97	11.20 ± 0.79	21.5	0.004
	NTG	10.71 ± 1.14	9.64 ± 1.08		
Mixed	TG	22.70 ± 2.31	24.70 ± 1.95	22.5	0.008
	NTG	22.69 ± 2.90	21.54 ± 2.30		
Total	TG	61.15 ± 5.99	66.60 ± 4.01	*T*-test: *t* = 4.775, df = 21	<0.001
	NTG	61.85 ± 5.18	58.00 ± 5.32		

### Pass vs. Fail

**Figure 2:**
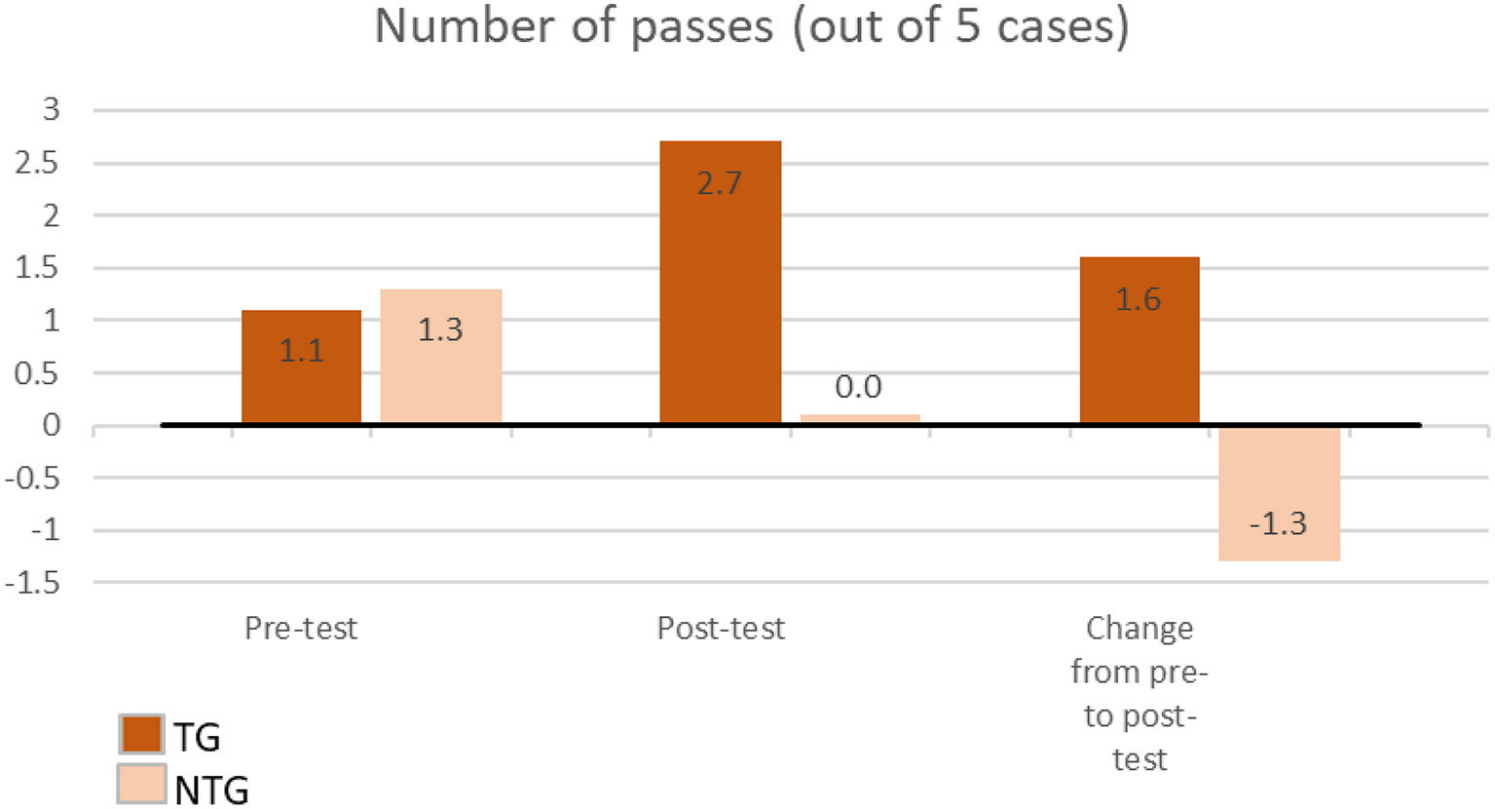
Pre- and Post-test passes for students, assessing the change between TG and NTG.

A Mann–Whitney *U*-test showed a significantly greater number of cases passed overall in the TG compared to the NTG (*p* < 0.001). Out of a possible 5, the average number of passes post-test was 2.7 (range 0–5) in the TG and 0 in the NTG. Comparing the number of cases passed between TG and NTG found a significant difference pre- to post-test (*p* < 0.001), with an average increase of 1.6 passes for TG and average decrease of 1.3 passes for NTG (Figure 2).

### Error Types

Common errors included: prescriber's qualifications not documented, no streamline code, no units on the drug strength, the formulation of the medication not clear, quantity not written in words and items not numbered for S8 case and items not on separate scripts for the mixed case.

## Discussion

This study investigated if prescribing skills of final year medical students changed following a structured pharmacist-led education intervention. Those randomised to receive the educational intervention significantly improved their prescribing skills whereas those randomised to standard medical ward rotation training were observed to exhibit significantly worsened prescribing skills. The change in scores between the randomised groups were consistent regardless of comparing overall or individual case scores. It is likely that improvements in the intervention group (TG) were due to the prescribing-specific education provided by the pharmacists. The apparent decrease in skills in the control group (NTG) was concerning and potentially a result of inconsistent prescribing-specific experiential teaching and learning. Previous studies have demonstrated that instilling poor skills and habits amongst medical students early in their clinical development is avoidable with appropriate training by informed educators (18, 19).

The salient result of this study is the pass/fail rate representing the number of students' cases which complied with all legal and safety requirements. The overall number of cases passed was significantly more in the TG compared to the NTG. We extrapolate that an increased number of legal and safe prescriptions may reduce future adverse events and costs to the health sector, though assessment of such medium-long term impacts was not possible in this pilot study. In a fourth-year medical students' response to Newby et al., they believe that positive habits in prescription writing would form with pharmacist-led education being established as a longitudinal theme, revised and regularly updated (20).

Prescribing errors relating to legal and safety requirements of a prescription are common, occurring at a rate of four to five per patient on paper-based prescriptions (1). Increased education on prescription writing for medical students may decrease the number of errors and therefore decrease these adverse events, improving patient outcomes (14). Many of the errors identified in this study originated from lack of knowledge and familiarity around legal requirements of prescriptions in Queensland. For example, requesting a 7-day supply of a controlled drug in the questionnaire assessed knowledge of safety specifically for a Queensland regulatory context. Whilst the PBS allows 14 days supply under federal regulation; students were required to supply a quantity lasting 7 days and those whose scripts reflected the PBS quantity were marked down. Our reasoning was that safe script writing must be a component taught with the legal requirements of prescribing within the specific local jurisdiction in which it is hierarchically applied.

There is no global standard for prescriptions as per the WHO GGT (21). However, it states the most important requirement is for the prescription to be clear and legible. Furthermore, it is the legal duty of a prescriber to produce an unambiguous prescription (21). All the prescriptions assessed in this study were legible. Good legibility was anticipated as the assessments were performed in a controlled environment without outside stimuli or time pressures that heuristically may otherwise influence student handwriting abilities.

Most students in this study sat an additional education session during their medical rotation through the palliative care centre. This education consisted of a specialist practicing physician educating students specifically on “controlled drug” prescription writing. Comparing educators was not the purpose of this study. However, a number of articles have demonstrated pharmacists have a positive influence on prescribing behaviours of prescribers (18, 19, 21, 22). Given that all students, except one, received this education from a prescribing physician it is unlikely to represent a confounder to our findings. Further, given that we observed superior prescribing skillset performance only amongst the group randomised to receive pharmacist-led education, we conclude the pharmacist-led education on script writing was effective in an additive compared to the alternative model of physician education. It has previously been observed that physicians in the role of educators may at times overlook some required knowledge and skills (18, 19). We hypothesise that students potentially felt more comfortable asking a pharmacist questions rather than a senior colleague to spare criticism or judgement given the traditionally hierarchical nature of medical workforce structures. Tittle et al. identified that students highly regarded pharmacists as teachers, finding them knowledgeable and approachable (22). The findings in this study support Tittle et al.'s conclusions.

Newby et al. also found that pharmacist-led education to medical students had a positive influence on their learning (3). Interprofessional learning creates an environment similar to professional practice which may prepare students for the holistic care provided to patients in a team-based scenario. A clinical pharmacist presented the education session using CBL. CBL offers an ideal opportunity to learn relevant material and apply knowledge in different situations. Our results demonstrate CBL and establishing a relationship with pharmacists allowed the TG to learn during the intervention, apply their knowledge and develop further during the subsequent 3-week placement, leading to improved results at post-test.

Engraining positive prescribing habits and teaching students through CBL on the legal and safety requirements of prescription writing during their final year of medical school may encourage short-term retention of knowledge as students' progress toward their intern year. Further research using the same students in a yearlong follow-up period will be required to confirm this hypothesis and begin to explore medium- and longer-term impacts. The engaging format used by the clinical pharmacist was another strength of this study. The session was formatted to be interactive, avoiding long learning segments and encouraging students to participate in practice prescription writing. Using the research design of a randomised controlled trial and having a control group to compare the pre- and post-results reduced bias and was a strength of this study. Limitations of this study were that it was only performed at a single site, using a small cohort of students, with one education session. The characteristics of those not recruited were not collected and assessment marking was not blinded due to time constraints and available pharmacists. A larger cohort and consistent student follow-up will be necessary to corroborate the study results, including delivery by multiple different pharmacist educators with sufficient powering to overcome confounding effects. Blinded marking of the assessment should be used in future studies to remove the risk of bias in the measurement of the outcome.

Future studies should address these limitations and follow-up students in their intern year to assess retention and application of knowledge in real world settings. For example, one model may be to conduct the intervention in a simulated clinical environment mimicking a real-life ward setting to replicate factors that contribute to prescribing errors. We propose future investigation and research should incorporate clinical knowledge into assessment, as this can greatly influence a student's and subsequent prescriber's ability to generate effective, appropriate and safe prescriptions.

## Conclusions

This study demonstrates that education to final year medical students by a clinical pharmacist on the legal and safety factors of prescriptions is beneficial in terms of their prescribing skills over a short time frame. We propose pharmacist-led education models be further studied and investigated to assess potential for incorporation into medical school curricula. Further improvement may be needed in the future on standards put forth on prescription writing by such institutions as the Australian Medical Council and Medical Board of Australia. Elaboration should be made on what, and how, teaching is delivered and assessed and a requirement to have handwritten prescriptions compliant with local legislation. We postulate that proximity of education to the intern year aids retention of knowledge, as students may be more interested in the education sessions, knowing they will use the skills the following year. Results showing poor prescribing skills acquired solely from standard ward rotation experiential and peer learning (NTG) suggests that experienced prescribers may also benefit from future pharmacist-led prescribing educational models.

## Data Availability Statement

The datasets presented in this article are not readily available because data set has not been anonymized. Requests to access the datasets should be directed to sophie.mokrzecki@health.qld.gov.au.

## Ethics Statement

The studies involving human participants were reviewed and approved by Townsville Hospital and Health Service Human Research and Ethics Committee and endorsed by the James Cook University Townsville Ethics Committee. The patients/participants provided their written informed consent to participate in this study.

## Author Contributions

SM created the concept and design of the research project and conducted the intervention, analysed and interpreted the data. Writing of the manuscript, and drafting and coordination of edited versions were managed by SM. TP and AM critically analysed the paper which was highly important for the intellectual content of the paper. TP assisted with analysis and interpretation of data, while AM assisted in the guidance and direction of the paper. SP assisted SM in finalising the research design and conducting the intervention, acted as a second reviewer of data for SM, and provided guidance for data presentation. SP also provided critical analysis when drafting the paper and offering different intellectual content. All authors have provided final approval for publishing and was held accountable for all aspects of the work and ensure accuracy and integrity of each part of work.

## Conflict of Interest

The authors declare that the research was conducted in the absence of any commercial or financial relationships that could be construed as a potential conflict of interest.

## Publisher's Note

All claims expressed in this article are solely those of the authors and do not necessarily represent those of their affiliated organizations, or those of the publisher, the editors and the reviewers. Any product that may be evaluated in this article, or claim that may be made by its manufacturer, is not guaranteed or endorsed by the publisher.

## Appendices

**Appendix 1:** All possible errors identified on a prescription.

1. Patient factors
  - Name
  - Address
  - [Additional to above] Date of birth on a controlled drug prescription

2. Prescriber factors
  - Name
  - Qualifications
  - Signature
  - NOTE: The place of practice address was already pre-printed on the prescription copy

3. Drug factors
  - Name of drug
  - Strength of drug (with unit e.g., mg)
  - Clear directions for a pharmacist to dispense and a patient to understand
  - Quantity in figures
  - Quantity in words and figures (for a controlled drug)
  - Indication in repeats box (either crossed out/nil/0 or a number generally coinciding with PBS written)
  - Streamline if required

4. Legal factors
  - Date the script was written
  - If two controlled drugs on one prescription, each drug is numbered
  - If two controlled drugs on one prescription, a line under the last item
  - Where a drug from another case is written on the safe prescription as another case, it must still be legal (eg. schedule 4 prescriptions only together, not written with a schedule 8 medication)

5. Safety factors (variable not calculated into error types but noted)
  - Clear and legible
  - No ambiguity
